# Feasibility and Accessibility of a Tailored Intervention for Informal Caregivers of People with Severe Psychiatric Disorders: a Pilot Study

**DOI:** 10.3389/fpsyt.2017.00178

**Published:** 2017-09-21

**Authors:** Shyhrete Rexhaj, Claude Leclerc, Charles Bonsack, Philippe Golay, Jérôme Favrod

**Affiliations:** ^1^Institute of Higher Education and Research in Health Care, University of Lausanne, Lausanne, Switzerland; ^2^Community Psychiatry Service, Department of Psychiatry, University Hospital Centre, Lausanne, Switzerland; ^3^School of Nursing Sciences, La Source, University of Applied Sciences and Arts of Western Switzerland, Lausanne, Switzerland

**Keywords:** nursing, caregivers, program development, psychiatric disorders, recovery

## Abstract

**Objectives:**

This study aimed to assess the acceptability and feasibility of a new tailored intervention for informal caregivers: the *Ensemble* (Together) program.

**Methods:**

An open pre–post within-subject comparison pilot study was conducted. Twenty-one informal caregivers completed the five-session *Ensemble* program. Two measurement tools were used: The Brief Symptom Inventory (BSI) and the Life Orientation Scale (LOT-R).

**Results:**

The results showed that informal caregivers were in need of individual support and were ready to participate in the *Ensemble* program independent of the patient’s diagnosis or stage of illness. The participants were very satisfied, and 95.4% completed the program. The preliminary results also showed that in five sessions, informal caregivers’ Global Severity Index measured by the BSI and their optimism about their future (measured by the LOT-R) were significantly improved.

**Conclusion:**

This pilot study provided preliminary results concerning the feasibility and acceptability of the tailored *Ensemble* program and indicates the need for a randomized trial. The *Ensemble* program is appropriate for both the acute and chronic phases of disease. Individualized brief and useful interventions for informal caregivers may provide more positive outcomes in care.

## Introduction

Within the mental health system, community-based care is considered to promote patients’ recovery and to facilitate access to care (1, 2)*;* however, various actors within the health system, including informal caregivers, must work together to ensure that an appropriate health plan is reached (3). In this context, informal caregivers provide important support not only during remission but also in acute episodes of illness (4). Informal caregivers are significant others who feel concerned by and provide support to the patient (5). The scientific literature has shown that the demands of the informal caregiver role can have negative consequences on a caregiver’s life. Specifically, recent studies have indicated that this role is related to a lower health state and reduced quality of life (6–8), which are associated with higher caregiver burden as well as poorer patient outcomes (6, 7, 9). Isolation and stigmatization can also negatively influence informal caregivers’ psychological health (10). Stigmatization is related to illness perceptions and caregivers’ coping strategies (11). The reasons for reduced access to professional interventions such as lack of available support, obstacles, time, and costs have also been described as factors potentially increasing caregivers’ experience of painful emotions (12), which Kaas et al. (13) suggested can affect their inclusion in a patient’s care plan.

Different psychoeducational approaches have been used by health professionals to support caregivers of people with severe psychiatric disorders. Authors in this field have provided the following recommendations: (a) various psychoeducation programs target the prevention of relapse in patients and improve the psychosocial and family “functions” associated with schizophrenia and bipolar disorder (14, 15); (b) intervening at the onset of disease to improve the impact of an intervention is currently recommended (16); and (c) it is important to take action while the patient is in an acute phase of illness to better support informal caregivers’ emotional needs (17). Providing emotional support for informal caregivers at the beginning of an illness in particular is recommended because this is a critical phase. The experience of painful emotions, such as denial of disease and feelings of being overwhelmed or shocked (12) can have serious consequences on their health and on the patient’s recovery (17, 18). Previous studies have also identified that informal caregivers need tailored knowledge about the patient’s illness, clarification about their roles and responsibilities, better control over their own life, and effective collaboration with health professionals (12, 17, 19–23). Additionally, scientific data recommend adjusting caregivers’ support according to the phase and severity of illness, as well as the caregiver’s sociodemographic characteristics (17). Most of the interventions published in the literature focus on the ill family member and its support but not on the specific needs of the informal caregivers as the core intervention. To our knowledge, no individual program targeting caregivers’ needs exists, except the study of Lobban and colleagues (24). To reduce the gap between scientific recommendations and actual practice, a new tailored intervention called *Ensemble* (i.e., together) was developed. Ensemble assesses the needs of informal caregivers and provides a tailored brief intervention.

### Aims of the Study

The current study aimed to assess the feasibility and acceptability of the *Ensemble* program, a brief intervention for informal caregivers of people with severe psychiatric disorders.

## Materials and Methods

### Design and Participants

A pilot study with an open pre–post within-subject comparison design was used to evaluate the feasibility and acceptability of an intervention aiming to support the target population of informal caregivers of people suffering from severe psychiatric disorders. This population included caregivers who lived in the community and wanted professional support for needs associated with having a relative with a mental disorder. The term “caregivers” included all people who were concerned about the patient. Caregiver, informal caregiver, and family caregiver are all terms used to describe family members, friends, or significant others who support people with severe psychiatric disorders and believe that they fulfill a caregiving role. The inclusion criteria were as follows: being at least 18 years old, living in the Lausanne catchment area, speaking French, having an adult relative suffering from a psychiatric disorder (with or without an established diagnosis) and having the capacity to agree to participate in the project. No exclusion criteria were established. The participants in this study were caregivers and were informed by an information sheet about this research project. This informational notice was available in the waiting rooms of early and community psychiatric care services and was also published on different internet sites for informal caregivers in need of support, including informal caregivers of patients who were not diagnosed or followed by selected psychiatric care services.

### Ethical Considerations

The research protocol received full authorization from the local ethics Committee in Switzerland (Commission cantonale (VD) d’éthique de la recherche sur l’être humain). As per the ethical approval, written informed consent was not obtained but participants were informed that the program Ensemble was being offered for the first time and by agreeing to follow it, they agreed that their data should be used for research purposes. An information sheet was distributed to them in which they were informed of their right to withdraw from the study at any time if they wished without any explanation.

### Outcome Measures

The following data and self-report scales were used in the pre- and posttests. A psychologist not involved in the intervention who was trained in the administration of the instruments collected the data. The average time that the participants needed to complete the scales was 20–30 min. In the posttest, the participants were seen by the same psychologist as in the pretest, who conducted a semi-structured interview to assess their satisfaction with the program. A paper and pencil, four-point Likert scale was then fulfilled by the participant.

The following sociodemographic data were collected though an interview: gender, age, education level, professional activity, the nature of their relationship with the patient, whether they lived with the patient, number of close contacts, previous requests for help, patient’s diagnosis according to the caregivers if known, and the duration of illness. The reasons for requesting help were also gathered during this interview.

#### The Brief Symptom Inventory (BSI), French Version: Informal Caregivers’ Psychological Health Status

The BSI is a brief psychological self-report symptom scale that includes 53 items (25). These items are organized into nine primary and clinically relevant symptom dimensions: (1) somatization, (2) obsessive–compulsive, (3) interpersonal sensitivity, (4) depression, (5) anxiety, (6) hostility, (7) phobic anxiety, (8) paranoid ideation, and (9) psychoticism. This scale has also three global distress indices: the Global Severity Index (GSI), the Positive Symptom Distress Index and the Positive Symptom Total. The BSI can be used for adult or adolescent patients and for individuals without disease. The BSI scale has been used in a variety of clinical and counseling settings as a screening tool for mental disorders and as a method of measuring symptom reduction (26–29). It has also been used with informal caregivers to assess their psychological health status (30, 31). In this study, the GSI is used as the main outcome measure because it represents the mean of the nine primary symptom dimensions and is more sensitive than the two other global indices (25). Higher GSI scores would indicate a greater effect on informal caregivers’ psychological health. The validation of the French BSI scale indicated good internal consistency for the GSI score (α = 0.91) (32).

#### The Life Orientation Scale (LOT-R), French Version: Optimism

The LOT-R scale, developed by Scheier et al. (33), measures an individual’s optimism regarding a given situation. This scale is designed to measure the adaptive strategies correlated with well-being. The LOT-R has been translated and validated in French, with good psychometric proprieties (internal consistency α = 0.76) (34). The LOT-R is a self-administered scale used to evaluate optimism versus pessimism. This scale includes 10 items; three measure optimism, three measure pessimism, and four function as fillers. The participants respond to each item on a 5-point Likert scale ranging from 0 (strongly disagree) to 4 (strongly agree); the four filler items are not included in the total score calculation. Higher scores suggest more optimism. Optimism has been shown to be negatively correlated with distress (35, 36) and to positively influence quality of life (37). In informal caregivers in particular, optimism promotes engagement in supportive programs (38), whereas pessimism leads to the use of avoidance strategies, which can predict informal caregiver burden (39). The LOT-R scale is a secondary outcome.

### Description of the Intervention

*Ensemble* (together) is a brief individualized intervention designed to promote the well-being of family caregivers who experience the effects of patients’ psychiatric disorders. The “Mapping Intervention Design (MID)” methodological framework (40) was used to develop an evidence-based intervention that focused on informal caregivers’ health promotion and recovery. The MID is performed in six steps: (1) needs assessment; (2) matrices (project plan); (3) program ideas; (4) program components and delivery channels; (5) program implementation; and (6) evaluation. Table 1 presents the principal results of the activities performed for each of these six steps.

**Table 1:** Ensemble program development based on a Mapping Intervention Design (MID) design (41).

MID steps	Sources of information or activities	Results
Step 1: needs assessment	*Literature review*: scientific recommendations regarding interventions for caregivers of patients with psychiatric severe illness *Context*: an inventory of existing interventions at the local level was performed. Then, recommendations from expert clinicians were obtained *Preferences of target population*: a focus group with six informal caregivers and three semi-structured interviews were conducted after presenting the results of the literature review, the inventory of existing interventions and the expert clinicians’ recommendations	The new intervention should be brief, individualized, and not specific to patient’s diagnosis to ensure early access for all informal caregivers
Step 2: matrices	Precise planning of project development steps. Different meetings with developers	The objectives of the *Ensemble* program were determined. The support provided must respond to caregivers’ unmet needs, painful emotions, and social resources
Step 3: program ideas	Integration of theoretical assumptions and scientific recommendations regarding the objectives of the program. The Neuman Systems Model, by Betty Neuman (42) and the Stage Model of Recovery, by Andresen et al. (43) were integrated as the theoretical framework	The structure of the program was designed, and all necessary material was evaluated
Step 4: program components and delivery channels	Final components of the program were determined with developers and informal caregivers. Two informal caregivers tested the final version, and their recommendations were incorporated. The program was delivered by a specialist nurse	Program components were selected, and large communication efforts were performed (internet, conferences, and papers)
Step 5: implementation	Recruitment of the target population. Promotion of the program. Supervision of the clinical party by an expert psychiatrist	The program was conducted
Step 6: evaluation	Different measures were selected to assess the program’s effects on the informal caregivers: Health: *Brief Symptom Inventory* Optimism: *Life Orientation Scale* Satisfaction: *Semi-Structured Interview*	An open pre–post within-subject comparison design was selected. The results showed an improvement in selected measures. The participants were very satisfied with the intervention quality

The six-step MID method enabled the incorporation of not only scientific recommendations (14, 17, 19, 23) but also theoretical assumptions, which were developed by integrating the Neuman Systems Model (42) and the Stage Model of Recovery by Andresen et al. (43). This integrated theoretical framework showed that informal caregivers experience stress due to the patient’s psychiatric illness. This stress can affect their capacity to recover depending on their resources. Therefore, in the first session of the Ensemble program, the nurse assesses the caregiver’s individual variables and offers a positive environment to enable informal caregivers to be responsible actors. In the following sessions, the support provided is targeted to the informal caregiver’s resources, regardless of scientific recommendations. This process enables collaborative care. The program was adapted to our context as suggested by the MID methodological framework (44).

The *Ensemble* program aims to accomplish the following:

– Identify caregivers’ needs and difficulties, as well as the painful emotions induced by experiencing illness in one of their relatives
– Improve caregivers’ awareness of the available social support
– Recognize the implications of being a caregiver and share concerns related to this role
– Share the experience of being a caregiver with someone who has had similar experiences
– Identify methods that promote personal well-being such as problem solving or management of painful emotions
– Plan next steps by targeting the available support structures according to caregivers’ unmet needs

Five sessions between the family caregiver and nurse (without the patient) are conducted. The same nurse conducted all the sessions at the more convenient place for the participant. The session take place at the nurse office, at participant home, or by Skype when participant was away. The session lasts 1 h once a week for 5 weeks.

#### Session 1: Assessment and Engagement

The first session aims to assess family caregivers’ needs in all life dimensions. This assessment is led through an interview using three clinical tools: (1) the Difficulties and Needs Self-Assessment Tool, (2) Painful Emotions Tool, and (3) Social Network Tool.

(1) The difficulties and needs self-assessment tool includes two independent scales, one focusing on difficulties and the other on support for unmet needs. Twenty-one areas of life are assessed that enable identification of priority problems and orientation of support according to the level of emergency. These 21 areas of life are organized into four life dimensions: life conditions, daily pragmatic activities, relationships, and health.
(2) The Painful Emotions Tool uses photos that reflect painful emotions such as guilt, judgment from others, loneliness, sadness, distress, despair, anxiety, helplessness, anger, confusion, and shame. The caregiver selects the painful emotions that are present in his/her life. The tool also assesses the frequency of the emotions. Consequently, the support provided is targeted to the caregiver’s most painful emotions.
(3) The Social Network Tool uses a network card that specifies the social resources available to the caregiver. This tool provides a graphic representation aimed at identifying the caregiver’s primary, secondary, and tertiary environment.

In the first session, it is essential to encourage the caregiver’s engagement and trust in the program. To ensure their engagement in the intervention processes, caregivers need to be welcomed, respected, and considered a partner. A compassionate attitude among nurses can also reduce stigmatization and caregiver isolation.

The approach used in this session aims to individualize the support for each caregiver (45).

#### Sessions 2, 3, and 4: Concrete Professional Support

The concrete support provided is adjusted according to the first assessment session. This support consists of three meetings that are designed to provide concrete assistance focused on hope and recovery and to help relatives perform the functions of an informal caregiver. The concrete professional support implemented is determined in collaboration with the caregiver. However, the following nursing actions are identified and often used during these three sessions depending on the caregiver’s needs.

##### Nursing Actions

###### Knowledge

Knowledge provide information about caregiver’s health, patient’s mental illness and useful information about care services. Informal caregivers may also be interested in knowing more about patients’ rights and about their own responsibilities and opportunities.

###### Coordination and Coping Strategies

Coordination and coping strategies studies have shown that caregivers face various problematic situations when caring for patients with mental illness [accompanying them to various meetings with professionals if they refuse; searching for strategies to reduce intra-family tension; helping them with daily activities and supervising their use of medication (39)]. To address these situations, problem-solving training has been applied to help caregivers identify and define the problem, search for different resolutions, assess their consequences (advantages and disadvantages), and choose the most effective resolution (46).

###### Reduced Stigmatization and Isolation

Illness perceptions are essential to reducing stigmatization and isolation among informal caregivers. Sharing caregiver’s illness perceptions is also critical to adapting the information provided to the culture and context, which can ensure effective nursing actions (5). If the participant expressed the need to meet another informal caregiver or expressed a feeling of loneliness, a meeting between the participant and a peer was organized. The nurse can be present or not depending on the participant’s needs.

###### Painful Emotions

The participants performed cognitive or practical exercises including relaxation-meditation to help them manage painful emotions. A practical exercise called “crisis of calm,” which was developed by Cungi and Deglon (47), can be trained in 3 min during one of the three session and practiced by the caregiver every day thereafter to manage painful emotions or to relax.

#### Session 5: Plan the Future

The last session aimed to review all the sources of professional support available as well as to help the participant become aware of the change in needs assessed at the beginning of the program. It is also essential to plan next steps in this session regardless of the caregiver’s accomplishments.

### Data Analysis

Data were analyzed using “IBM SPSS Statistics^®^ Version 20.” Descriptive statistics for sociodemographic data were used. Pre- and post-outcome measures regarding the relative’s health based on the BSI and optimism according to the LOT-R were analyzed. To determine the potential effect of the intervention, all participants had to complete the *Ensemble* program. Comparisons between the selected measures pre- and post-intervention were performed. Paired *t*-tests were conducted to describe the differences between measures pre- and post-intervention.

## Results

### Sample

Twenty-two participants started the program, and one dropped out after three sessions. The results in Table 2 show that 21 informal caregivers completed this study: 15 women and 6 men. There were more parents (66.7%) than other family members. Completed education level was a university-level education for 38.1% of the participants. The majority of the sample were professionally engaged (71.4% were salaried or were their own employer and 4.8% were students). Informal caregivers had daily close contact with the patient for 61.9% of the sample, and 52.4% lived under the same roof with the patient. Furthermore, 61.9% of participants had benefited from previous professional support but were interested in participating in the *Ensemble* program. Seven participants wanted to facilitate research and to benefit from individualized support but did not provide specific expectations. The need to understand the caregiver’s role and the illness to better support their ill relative was also mentioned as a reason for participation. However, most informal caregivers had many reasons for their involvement in *Ensemble* program; they identified several unmet needs and painful emotions. The patients whose informal caregivers participated in the *Ensemble* program suffered from various severe psychiatric disorders. Schizophrenia and depression were the two most common diagnoses mentioned by the participants, followed by bipolar and anxiety disorder. The mean duration of patient’s disease was more than 3 years; however, 42.9% of patients were at their first episode and 33.3% had an illness duration less than 1 year.

**Table 2:** Participants’ sociodemographic characteristics (*N* = 21).

Age, mean (SD)	47.52 (14.98)
Sex, *N* (%)	
Female	15 (71.4)
Male	6 (28.6)
Relationship type, *N* (%)	
Mother/father	14 (66.7)
Sister/brother	3 (14.3)
Wife/husband	2 (9.5)
Daughter/son	2 (9.5)
Completed education level, *N* (%)	
Compulsory education	4 (19.0)
Apprenticeship	4 (19.0)
High school graduate	2 (9.5)
School profession, trade, normal, technical	3 (14.3)
University	8 (38.1)
Professional activity, *N* (%)	
Salaried or own employer	15 (71.4)
Pensioner	2 (9.5)
Unemployed	3 (14.3)
Student	1 (4.8)
Living under the same roof as patient, *N* (%)	
Yes	11 (52.4)
No	10 (47.6)
Frequency of close contacts, *N* (%)	
Daily	13 (61.9)
Weekly	7 (33.3)
Monthly	1 (4.8)
Previously requested help, *N* (%)	
Yes	13 (61.9)
No	8 (38.1)
Obtained first individual help as a caregiver	
Yes	21 (100)
No	–
Reason for requesting help, *N* (%)	
Participate in research	7 (33.3)
Manage the caregiving role	6 (28.6)
Better understand the disease	5 (23.8)
Gain professional support	2 (9.5)
Support patient	1 (6.7)
Number of reasons for the request, *N* (%)	
One reason	7 (33.3)
Several reasons	14 (66.7)
Patient’s diagnosis according to the caregiver, *N* (%)	
Schizophrenia	13 (61.9)
Depression	4 (19.0)
Bipolar disorder	2 (9.5)
Anxious disorder	2 (9.5)
Duration of illness, *N* (%)	
Less than a year	7 (33.3)
Between 1 and 2 years	2 (9.5)
Between 3 and 10 years	9 (42.9)
More than 10 years	3 (14.3)

### Comparison between Pre- and Post-Tests

Table 3 shows that informal caregivers’ health significantly improved on Global Severity Index of the BSI [*t* = −2.149, df (20), *p* = 0.044], with a Cohen’s *d* effect size of 0.47. As secondary outcome, Optimism measured with LOT-R, Life Orientation was significantly improved [*t* = −2.575, df (20), *p* = 0.018] with an effect size of Cohen’s *d* of 0.58.

**Table 3:** Pre- and post-intervention differences in 21 informal caregivers (*t*-test; *p*-value).

	Pre-test mean (SD)	Post-test mean (SD)	*t* (df); *p*	Cohen’s *d*
**Brief symptom inventory**				
Global Severity Index	0.72 (0.52)	0.53 (0.58)	*t* = 2.149 (20); *p* = 0.044	0.47
**Life orientation**				
Optimism	15.52 (3.47)	17.43 (3.95)	*t* = −2.575 (20); *p* = 0.018	0.58

### Participants’ Satisfaction

Family caregivers evaluated their satisfaction with participation in the *Ensemble* program. Table 4 shows that family caregivers were satisfied to very satisfied with the quality of the *Ensemble* program. There were also satisfied to very satisfied with the timeliness of the first appointment and the clarity of the explanations at inclusion, information received during the first visit and explanations regarding the research project and the *Ensemble* program.

**Table 4:** Participants’ satisfaction with the Ensemble program.

	Mean (SD)	Median (min–max)
**Welcome and information during the first contact**		
Fast and clear contact	4.00 (0.00)	4.00 (4.00–4.00)
Information received during the first appointment	3.72 (0.56)	4.00 (3.00–4.00)
Explanations of the research project and the intervention	3.67 (0.48)	4.00 (3.00–4.00)
**Quality of the intervention**		
The proposed intervention corresponds with my needs	3.81 (0.40)	4.00 (3.00–4.00)
The availability of the nurse corresponds with my expectations	3.95 (0.22)	4.00 (3.00–4.00)
The intervention helped me feel supported and listened to	4.00 (0.00)	4.00 (4.00–4.00)
I felt that the intervention was beneficial and comforting	3.86 (0.36)	4.00 (3.00–4.00)

*4, very satisfied; 3, satisfied; 2, a little satisfied; 0.1, dissatisfied*.

## Discussion

This study examined the feasibility and acceptability of the *Ensemble* program, which aims to support informal caregivers of patients with severe psychiatric disorders. The five-session *Ensemble* program provides informal caregivers targeted support to address their specific unmet needs, emotions and social resources. This pilot study showed that this program was easy to implement: 21/22 (95.4%) participants completed all sessions. Only one participant (a mother) stopped participating in the program, discontinuing after three sessions because she had newly diagnosed advanced cancer and needed time for her care. As indicated by the satisfaction measures, the participants were very satisfied with the program, demonstrating its acceptability.

The results also showed that after five sessions, the 21 participants’ psychological health status was better than at pre-test. These findings highlight that informal caregivers are at risk of developing psychological problems compared to non-clinical populations; for example, their GSI score pre-test (0.72) was higher than that of a British community sample (0.44) (48) and lower than that of a British outpatient sample (1.65) (49). At the end of the *Ensemble* program, the participants were also more optimistic about their future. This new program showed that we can promote informal caregivers’ health, which could have a positive impact on the persons they care for. This program was the first individual intervention to use caregivers as the entire participant population. Furthermore, this tailored approach used an MID methodological framework, which promoted the informal caregiver’s role; therefore, the informal caregiver was considered a key partner and actor who was responsible for his/her own health.

Additionally, although individualized interventions have been recommended (22, 50), few studies have presented individualized interventions for informal caregivers that could be compared with the current pilot study. Lobban and colleagues (24) reported a self-management program for relatives of people with recent-onset psychosis; the results show lower distress and better capacity to adapt. The differences between the *Ensemble* program and the self-management program developed by Loban and colleagues (24) are as follows: (1) in *Ensemble*, informal caregivers receive specific support regarding their unmet needs, painful emotions and social resources and (2) the *Ensemble* program is not specific to a diagnosis. Regarding the first difference, informal caregivers can thus choose the domain in which the nurse helps them. Informal caregivers can sometimes have difficulties identifying painful emotions; accordingly, for the first assessment meeting, the nurse provides a secure environment to explore and manage these emotions. Regarding the second difference, informal caregivers can access tailored support without waiting for the patient’s diagnosis. Indeed, clinicians need time to assess patients, and psychiatric diagnoses sometimes evolve. Even when patients are diagnosed with the same disease, the severity of consequences and the adaptation strategies need to be considered in a differentiated manner (51).

Another study presented a family led program called “*The Journey of Hope*” that was independent of patient’s diagnosis (52). The results of that study showed an improvement in illness knowledge and reduction in information needs but did not indicate a direct impact on informal caregivers’ health outcomes. The tailored *Ensemble* program supported informal caregivers in their specific needs. For example, siblings of patients are often overlooked in the clinical and research domains, even though they play a significant role in their brother’s or sister’s life (53). Few specific programs for siblings have been developed (54). The *Ensemble* program seems well suited to support siblings; additionally, the two sisters and the brother who participated in our study were very satisfied with the flexibility of the nurse.

Only group psychoeducation programs that are specific to patient’s diagnosis are implemented in our setting, and the support is similar for all informal caregivers. These programs have their advantages, but many informal caregivers have to first wait for the patient’s diagnosis to be determined. The *Ensemble* program is a good alternative and promotes well-being as soon as symptoms first appear. Studies have shown that isolation and stigmatization (10, 55) can increase informal caregivers’ burden and difficulties helping the patient access psychiatric services. Supporting informal caregivers in this phase could not only promote caregiver well-being but could also encourage patient involvement in care. Some informal caregivers need specific psychiatric care for their own health, and their participation in the *Ensemble* program could lead to earlier detection of disorders and quicker referral to appropriate services. These benefits could reduce the costs of health, which are very high, especially for unipolar depression (56). The *Ensemble* program is appropriate in both acute and chronic phases of disease because the support is targeted to the participant’s needs. Informal caregivers can access support when they need it. Patient recovery is also known to be time consuming, and patients can have acute phases of illness during this process (57)*;* therefore, some of the participants wanted tailored support even if they had previously received another professional help service. This finding is in accordance with scientific recommendations to support informal caregivers depending on the phase and severity of the illness (17, 22). The *Ensemble* program was appreciated by all the participants, regardless of the patient’s diagnosis or recovery step, and ensured that the support provided was targeted to informal caregivers.

### What the Study Adds to the International Evidence

The tailored *Ensemble* program aims to support informal caregivers of patients with severe psychiatric disorders as soon as they are in need. On the primary outcome, the participants showed significant improvements in psychological health status measured by the GSI based on the BSI scale. Informal caregivers were also more optimistic regarding their future at the end of the program as secondary outcome. These favorable outcomes should be studied in future randomized trials. The participants were very satisfied with the intervention and the attrition rate was very low.

### Limitations

This study had several limitations. It was a pilot study without a control group, and the number of participants was small. The assessor, although not included in the intervention, was not blind to the study objectives. In this sample, men were less represented than women. An experimental study on the *Ensemble* program is needed. It would also be interesting to integrate other patient variables such as their functioning, symptoms and recovery levels. The results of this study allow calculations of the sample size needed for a controlled study.

### Implications for Practice

There is a lack of intervention focusing on the specific needs of informal caregivers of patients suffering from a severe psychiatric disorder. The development of the program Ensemble and this pilot study are a first step to bridge this gap. The *Ensemble* program is appropriate for both acute and chronic phases of disease because the support is targeted to the caregivers and is brief, tailored and useful.

## Ethics Statement

The research protocol received full authorization from the local ethics Committee in Switzerland (Commission cantonale (VD) d’éthique de la recherche sur l’être humain).

## Author Contributions

SR conceptualized the research and the program Ensemble, acquired, analyzed, and interpreted the data, and drafted the first version of the manuscript. PG contributed to the data analyses. CL, CB, and JF gave a substantial contribution to the analysis and interpretation of data and critically revised the article for important intellectual content. All the authors approved the final version for publication. All the authors agreed to be accountable for all aspects of the work by ensuring that any questions related to its accuracy or integrity can be appropriately investigated and resolved.

## Conflict of Interest Statement

The authors declare that the research was conducted in the absence of any commercial or financial relationships that could be construed as a potential conflict of interest.
